# Statin Use Ameliorates Survival in Oral Squamous Cell Carcinoma—Data from a Population-Based Cohort Study Applying Propensity Score Matching

**DOI:** 10.3390/biomedicines11020369

**Published:** 2023-01-27

**Authors:** Steffen Spoerl, Michael Gerken, René Fischer, Silvia Spoerl, Christian Kirschneck, Stefanie Wolf, Juergen Taxis, Nils Ludwig, Niklas Biermann, Torsten E. Reichert, Gerrit Spanier

**Affiliations:** 1 Department of Cranio-Maxillofacial Surgery, University Hospital Regensburg, 93042 Regensburg, Germany; 2 Tumor Center, Institute for Quality Management and Health Services Research, University of Regensburg, 93053 Regensburg, Germany; 3 Department of Otorhinolaryngology, University Hospital Regensburg, 93042 Regensburg, Germany; 4 Department of Internal Medicine 5, Hematology/Oncology, Friedrich-Alexander University Erlangen-Nürnberg, 91054 Erlangen, Germany; 5 Department of Orthodontics, University Hospital Regensburg, 93042 Regensburg, Germany; 6 Department of Otorhinolaryngology, St. Elisabeth Hospital Straubing, 94315 Straubing, Germany; 7 Department of Plastic and Reconstructive Surgery, University Hospital Regensburg, 93042 Regensburg, Germany

**Keywords:** oral squamous cell carcinoma, OSCC, HNSCC, statin, hydroxymethylglutaryl CoA reductase inhibitor, cardiovascular disease, CVD, survival, propensity score matching, PSM

## Abstract

The anti-cancer properties of statins have attracted much attention recently, but little is known about the prognostic role of statins in oral squamous cell carcinoma (OSCC). In a retrospective approach, we analyzed a population-based cohort of 602 OSCC patients with primary curative tumor resection to negative margins and concomitant neck dissection between 2005–2017. Long-term medication with statins was correlated with overall survival (OAS) as well as recurrence-free survival (RFS) using uni- and multivariable Cox regression. Additionally, propensity score matching was applied to adjust for confounders. Statin use was present in 96 patients (15.9%) at a median age of 65.7 years. Statin treatment correlated with ameliorated survival in multivariable Cox regression in the complete cohort (OAS: HR 0.664; 95% CI 0.467–0.945, *p* = 0.023; RFS: HR 0.662; 95% CI 0.476–0.920, *p* = 0.014) as well as matched-pair cohort of OSCC patients (OAS: HR 0.691; 95% CI 0.479–0.997, *p* = 0.048; RFS: HR 0.694; 95% CI 0.493–0.976, *p* = 0.036) when compared to patients not taking statins at time of diagnosis. These findings were even more pronounced by sub-group analysis in the matched-pair cohort (age < 70 years). These data indicate that statin use might ameliorate the oncological outcome in primarily resected OSCC patients, but prospective clinical trials are highly recommended.

## 1. Introduction

Cardiovascular diseases (CVD) are a leading cause of mortality, a major contributor to disability and remain a common healthcare challenge worldwide. Globally, prevalent cases of CVD nearly doubled from around 271 million in 1990 to around 523 million in 2019, while the number of CVD deaths steadily increased from around 12.1 million to around 18.6 million in the same period of time [1,2]. The Framingham heart study started to enroll patients in 1948 aiming to obtain deeper insights in the epidemiology of CVD and to identify possible risk factors [3]. Over the years, this study provided deeper insights in the development and progression of CVD and introduced several risk factors, including smoking, elevated blood glucose, obesity, and hyperlipidemia [4]. Especially elevated blood cholesterol levels were considered as important risk factors, favoring pathological conditions leading to CVD [5]. In the early 1990s, cholesterol-lowering drugs were introduced, notably 3-hydroxy-3-methylglutaryl-CoA reductase inhibitors, which are clinically known as statins. Besides the profound beneficial aspects of statins in prevention and therapy of CVD, emerging interest of repurposing statins as potential therapeutics for other diseases which are dependent on, or influenced by, cholesterol metabolism exists [6]. Hereby the significance of statins had been investigated in numerous studies and tumor entities: with regards to solid malignancies, statin use was shown to have preventive effects and reduced the risk of patients developing neoplasms such as gastric, liver, esophageal or reproductive cancer [7]. However, the therapeutic value of statins in cancer remains controversial, precisely due to contrary findings for distinct cancer types: While statin use was shown to drastically increase survival of patients with advanced hepatocellular carcinoma [8], no beneficial aspect on outcome of patients with lung cancer and glioblastoma was observed [9,10,11].

While the preventive and therapeutic effects of statins are well characterized in most carcinomas, little is known about the prognostic effects of statin use in patients with oral squamous cell carcinoma (OSCC). The retrospective population-based study of Gupta et al., which included 1592 head and neck squamous cell carcinoma (HNSCC) patients, correlated statin use with a significant improvement of patients’ outcome [12]. This study included carcinomas from all anatomical sites of the head and neck region; however, statin use seemed to play an important role in patients with oral carcinomas, improving their overall and cancer specific survival (OAS and CSS, respectively). These results indicate the urgent need for validation and further characterizing the clinical effects of statins on OSCC. This is of particular importance, since OSCC is defined as a distinct tumor entity, featuring an interdisciplinary challenge in head and neck oncology [13]. Although combined treatment concepts of surgery, radiotherapy, chemotherapy, and immunotherapeutic approaches were constantly improved during the last decade, only minor effects on patient outcomes were observed with special regards to patients with advanced tumor stages [13].

Due to the controversially discussed role of statins in affecting survival of cancer patients and the urgent need for validating the promising existing results for statin use in HNSCC [12], the aim of this study was to retrospectively analyze the role of statins in outcome of primarily resected OSCC patients, using multivariable Cox-regression analysis and propensity score matching (PSM) in a population-based cohort study.

## 2. Material and Methods

### 2.1. Population-Based Approach

In this retrospective multicenter cohort study, adult patients with a primarily diagnosed OSCC residing in the region of Eastern Bavaria were analyzed. This area includes a representative German population of around 2.3 million people. The population-based dataset was kindly provided by the “Tumor Center—Institute for Quality Management and Health Services Research, University of Regensburg”, which represents a regional center of the Bavarian Cancer Registry. Clinical diagnostics and treatment took place at three different medical centers: the Departments of Cranio-Maxillofacial Surgery and Otorhinolaryngology of the University Hospital Regensburg and the Department of Otorhinolaryngology of the St. Elisabeth Hospital Straubing.

The complete population-based cohort included 702 OSCC patients. Since no patient younger than 50 years received statins, we excluded this age cohort a priori.

### 2.2. Patient Cohort

A total of 602 patients with primarily diagnosed OSCC were enrolled in the study (Table 1). All participants received resection in curative intention between January 2005 and December 2017 without application of any neoadjuvant treatment. Patients with a previous cervical lymph node dissection or any radiotherapy/radiochemotherapy due to prior HNSCC were excluded. All included patients had a tumor resection to negative margins and a concurrently accomplished cervical lymph node dissection based on clinical and radiologic examination.

Staging was performed according to the “TNM classification of malignant tumors” published by the Union for International Cancer Control (UICC) in its 7th edition [14].

Additionally, patient-specific demographic, histological as well as clinical data were collected, encompassing gender, age at diagnosis, positive history of smoking and alcohol abuse, acetylsalicylic acid and metformine use, anatomical site, extranodal spread, grading, lymphatic and vascular invasion, as well as application of adjuvant therapies. For each participant Charlson comorbidity index (CCI) was calculated as previously described and without taking OSCC into account [15,16]. Definite therapy recommendations were made by an interdisciplinary tumor board, including advice for adjuvant radio- or radiochemotherapy. Recurrent disease could either be diagnosed by radiologic evidence with clinical correlation or histologic confirmation by biopsy. Survival follow up data concerning recurrence-free survival (RFS) and OAS were gathered from medical records, death certificates, registration offices and the Clinical Cancer Registry of the Tumor Center—Institute for Quality Management and Health Services Research, University of Regensburg. Recurrences were derived from clinical reports and were defined as local or locoregional relapse and/or recurrence as distant metastases. 5-year OAS rates, RFS and cumulative recurrence rates were analyzed based on the date of resection until the first event. Statin use as well as acetylsalicylic acid and metformine prescription were withdrawn from archived digital and paper-based patients’ records. However, due to the retrospective acquisition of data, detailed timespan and dosage of preoperative statin, acetylsalicylic acid and metformine use was not available.

### 2.3. Statistics

Continuous data are described as means, median, minimum, and maximum values. Categorical data are expressed as absolute frequencies and relative percentages. Characteristics of patients were compared using two-tailed Student’s t test for continuous data in case of normal distribution, otherwise Mann–Whitney U test was applied. Pearson’s chi-square test was used for testing independence between categorical variables.

OAS and RFS were compared with the Kaplan–Meier method. The follow-up and survival period were right censored using 31 October 2020 as the cut-off date, rendering a mean follow-up of 6.6 years (median 6.2 years). Survival differences were tested for statistical significance by the two-sided log-rank test (Mantel-Cox); the level of significance was set to 0.05. To determine the impact of statin use and further covariables on survival, we performed uni- and multivariable regression analyses using Cox proportional hazard models. In multivariable analyses, the hazard ratio (HR) for dichotomous statin use (y/n) was adjusted for the covariables age at diagnosis, CCI, UICC stage, grading, lymphatic invasion and vascular invasion. The variables were included in multivariable analyses if *p*-values of univariable analysis were less than 0.100. Comorbidity was adjusted for via CCI, categorized in groups with at least one, two, three or more diseases and a group without any disease taken into account by the CCI, excluding OSCC. Hazard ratios and corresponding 95% confidence intervals (CI) were estimated and considered statistically significant when the CI excluded 1.0, and a two-sided *p*-value was <0.05. Additionally, we established a matched-pair cohort by applying a 1:3 propensity score nearest neighbor matching with caliper 0.2, balancing for the adjustment variables named above. All analyses were performed using IBM SPSS Statistics, version 26.0 (IBM Corp., SPSS for Windows, Armonk, NY, USA).

## 3. Results

### 3.1. Clinicopathological Data of Patients

This retrospective multicenter cohort study comprises data from 602 patients which underwent surgical resection of an OSCC to negative margins, including neck dissection. In a matched-pair cohort of 359 patients generated through PSM the prevalence of statin use at time of diagnosis was 15.9% (96 patients), detailed clinicopathological characteristics are listed in Table 1: Most patients were male (71.9%), mean age was 63.6 years (66.5 years in group of routine statin intake), additionally most patients had a positive anamnesis of alcohol and smoking abuse (65.7% and 75.2%, respectively). Predominant tumor localizations were the floor of mouth and the tongue (68.9% and 27.4%, respectively). By comparing the statin and the control subgroups, statin use was more prevalent in elderly OSCC patients (*p =* 0.003), in cases with comorbidities (*p* < 0.001), in patients with a history of alcohol abuse (*p =* 0.007), as well as in the subgroup of routine metformine or acetylsalicylic acid intake (*p =* 0.003 and *p* < 0.001, respectively). Furthermore, Pearson’s chi-squared test revealed diagnosed statin treatment to be less likely associated with application of adjuvant therapy (*p =* 0.019).

### 3.2. Effects of Statin Use on OAS and RFS Rates

To determine survival in OSCC patients after curative tumor resection, OAS, RFS as well as recurrence rates in patients with (statin subgroup) and without statin treatment (control subgroup) were analyzed. Table 2 displays results of uni- as well as multivariable Cox regression analyses in the complete cohort including all ages (*n* = 602).

**Figure 1:**
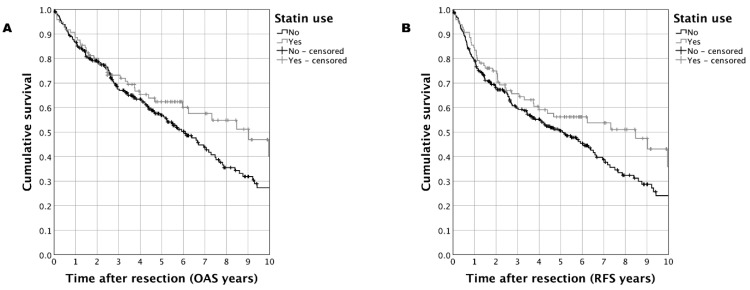
Survival in a matched-pair cohort of OSCC patients with age at diagnosis <50 years (*n* = 359) comparing patients taking statins to those patients without statin treatment: (**A**): Kaplan–Meier analysis for OAS (*p* = 0.099); (**B**): Kaplan–Meier analysis for RFS (*p* = 0.074).

**Figure 2:**
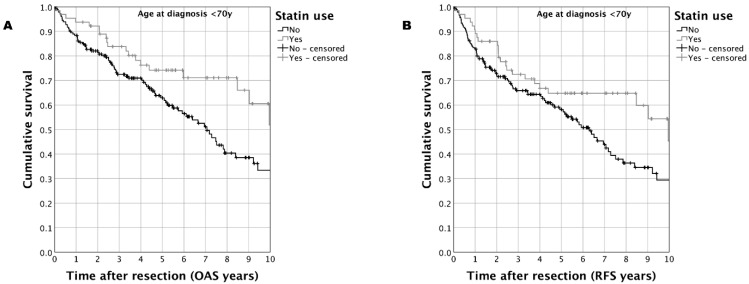
Survival in a matched-pair cohort of OSCC patients with age at diagnosis <70 years (*n* = 246) comparing patients taking statins at the time of diagnosis compared to those patients without statin treatment: (**A**): Kaplan–Meier analysis for OAS (*p* = 0.014); (**B**): Kaplan–Meier analysis for RFS (*p* = 0.032).

In the matched-pair cohort (*n* = 359) of OSCC patients including all ages, a trend towards improved survival was observed for OAS as well as RFS when performing Kaplan–Meier analyses (Figure 1). For the case group, a five-year OAS of 62.4% vs. 56.9% (case vs. control; *p* = 0.099) and a five-year RFS of 56.2% vs. 50.5% (case vs. control; *p* = 0.074) was determined. Sub-group analysis in matched-pair OSCC patients with an age <70 years substantiated these results: statin use was associated with a five-year OAS of 74.2% vs. 62.8% (statin vs. control; *p* = 0.014) and a five-year RFS of 68.8% vs. 58.1% (statin vs. control; *p* = 0.032) (Figure 2).

In univariable Cox regression analysis, statin use was not significantly correlated with OAS (HR 0.869; 95% CI 0.619–1.220, *p* = 0.418) as well as RFS (HR 0.839; 95% CI 0.610–1.155, *p* = 0.282) in the complete OSCC cohort comprising all ages (no PSM applied). However, in the complete cohort of OSCC patients <70 years (*n* = 459, no PSM applied) an improved OAS (HR 0.595; 95% CI 0.370–0.956, *p* = 0.032) as well as RFS (HR 0.656; 95% CI 0.429–1.003, *p* = 0.052) was observed. In the matched-pair cohort of patients <70 years revealed an even more pronounced effect for OAS (HR 0.537; 95% CI 0.325–0.887, *p* = 0.015) and RFS (HR 0.613; 95% CI 0.390–0.964, *p* = 0.034).

Data from multivariable Cox regression regarding statin use and oncological parameters in OSCC patients are mentioned in Table 3: Routine statin intake was significantly correlated with ameliorated OAS and RFS in the unmatched OSCC cohort (OAS: HR 0.664; 95% CI 0.467–0.945, *p* = 0.023; RFS: HR 0.662; 95% CI 0.476–0.920, *p* = 0.014) as well as in the entire propensity score-matched OSCC cohort (OAS: HR 0.691; 95% CI 0.479–0.997, *p* = 0.048; RFS: HR 0.694; 95% CI 0.489–0.976, *p* = 0.036). In line with the results of univariable survival analysis, statin use in propensity score-matched patients below 70 years was significantly and even stronger associated with ameliorated survival (OAS: HR 0.461; 95% CI 0.275–0.774, *p* = 0.003; RFS: HR 0.530; 95% CI 0.333–0.841, *p* = 0.007).

## 4. Discussion

Over 70 years ago, the sudden death of Franklin D. Roosevelt from CVD raised profound interests in obtaining a deeper understanding of the pathophysiology, risk factors, and especially prevention of CVD. As a main achievement of the consecutively initiated Framingham Heart Study, adverse effects of dyslipidemia and hypercholesterolemia were impressively brought up. Accordingly, lipid-lowering therapies were implemented in clinical routine, thereby statins currently represent one of the most and often prescribed drug group worldwide [17,18]. As a result of its common use, potential side-effects were frequently evaluated, including the question about a potentially carcinogenic effect of long-term statin intake [19]. In contrast to early publications, a growing body of evidence proposes anti-cancer potential of HMG-CoA inhibitors [18]. Thereby, the outcome of cancer patients as well as the risk to develop certain cancer entities were analyzed [18]. Especially for prostate cancer, a reduced risk for emerging aggressive tumor biological properties was shown in combination with statin therapy [20].

In OSCC, however, the data situation is quite insufficient, especially in terms of potential antitumor effects, and no literature with regards to comparable studies is currently available. Detrimental influences, leading to development and progression of OSCC, are ostensibly linked to regularly consumption of noxious agents such as alcohol, tobacco, and betel quid chewing [21], whereas on the other hand the prognostic significance of routinely taken drugs is still under debate. In our study, we analyzed the prognostic effect of statin use on OAS as well as RFS in OSCC patients. Multivariable Cox regression analyses revealed a profound survival benefit in primarily resected OSCC patients with routine statin intake compared to those without statin intake. Additionally, we established a matched-pair cohort by the means of PSM to adjust for covariables in our cohort, which confirmed the favorable results in survival analysis of statin intake in OSCC patients. Especially in patients younger than 70 years, who constitute the predominant part of the present cohort, statin use was significantly correlated with prolonged survival. As the first study performing PSM in a multicenter population-based OSCC cohort and thereby assessing prognostic effects of statin use in cancer, a comparison with other tumor entities might illuminate our results in a wider point of view:

In a meta-analysis including a maximum of 60 observational studies, the authors evaluated the effect of statin usage on oncological outcome parameters in various cancer entities. Amongst them, two studies were included regarding HNSCC. Yang et al., discussed several methodical limitations and in summary, data suggested that the use of statins may reduce cancer-specific mortality. Based on their results, statin use was not associated with reduced cancer progression-free survival [22]. Noteworthy, both HNSCC publications (Nielsen et al. [23] and Lebo et al. [24]) were excluded from that analysis.

In a large prospective cohort study with 1638 HNSCC patients Getz et al., proved that statin use may be protective for adverse outcome, particularly in an HPV-positive subgroup [25]. Although the literature entails inconsistent data about the prognostic value of statins influencing outcome of cancer patients, in vitro studies already evaluated potential cytostatic or cytoreductive properties of HMG-CoA inhibitors. With statins being characterized in vitro to induce apoptosis in several hematologic as well as solid tumor cell lines [26,27], the inhibitory effect of statins on distinct cellular pathways could be demonstrated: in HNSCC cell lines for instance, lovastatin inhibited epithelial growth factor receptor (EGFR) dimerization and internalization, with thereby presenting a further potential pharmacological cellular target [28]. Additionally, a potential role for statins in the mechanism of epithelial-to-mesenchymal transition (EMT) was proposed [6]. As a key mechanism in cancer progression, EMT entails several modified cellular properties, including enhanced migratory capacity, invasiveness as well as resistance to apoptosis [29]. In non-small cell lung cancer cells Atorvastatin was identified to potently inhibit EMT, proposing a further potential pharmacologically useable cellular mechanism in treating cancer patients [30].

Statins seem to exhibit beneficial immunomodulatory effects by reducing the number of circulating pro-inflammatory cytokines (e.g., Interleukin 6) in several entities [25]. In a murine lung cancer model, simvastatin had an anti-proliferative effect, it also proved immune tolerance-promoting value during tumor development [31].

A recent review by Lasgari et al., suggested that statins seem to have favorable effects on inflammatory, malignant and neurodegenerative diseases by inhibiting the mammalian target of rapamycin (mTOR) pathway [32]. Kansal et al., showed that statins enhance responses to immune checkpoint blockade in syngeneic murine models for head and neck cancer [33]. Kwon et al., demonstrated in a recent publication, that statins alone showed synergistic antitumor effects in HNSCC in vitro and in vivo. Furthermore, administration of statins in combination with cisplatin and anti-PD-1 immune checkpoint inhibition, enhanced the anticancer effect of the chemotherapeutic agent and potentiated the efficacy of immunotherapy. In addition, statins increased calreticulin and endoplasmic reticulum stress marker levels [34].

Moreover, a recent study indicated that statins might decrease the release of small extracellular vesicles by cancer cells, which are considered to promote cancer progression by initiating a pre-metastatic niche formation as well as promoting immunosuppression and angiogenesis [35,36,37].

Although in vitro results illuminated manifold cellular mechanisms in malignant diseases which might be clinically applicable for future treatment regimes, there are still remaining questions: Hereby not only the translational impact of statins on ameliorating survival of cancer patients is scrutinized, potential statin-associated side effects are clinically up for discussion. In our retrospective cohort study, no severe complications due to statin therapy, such as myopathy or transaminitis could be observed. A limitation in this regard is certainly the retrospective conception of this study. Basing on written and electronic patients’ records, a variable extent of accessible documentation certainly influences the study’s scientific significance. However, our analysis is the first study to address the question of potential protective effects of statin use solely in a large OSCC cohort. Given no observed severe complications, long-term statin use could be reported as safe and low in side effects during regular intake. In particular for OSCC patients below 70 years, daily statin use provided a striking and significant survival benefit. Ultimately, based on results of this multicenter propensity score-matched cohort study, randomized controlled trials should be conducted to further evaluate the prognostic potential of statins in OSCC.

## 5. Conclusions

In conclusion, the results of our study indicate that statins might improve outcome of OSCC patients which are selected for curative tumor resection. Conceived as a propensity score-matched retrospective study on a population-based cohort of primarily surgically treated tumor patients, statin use did especially affect OAS and RFS in patients younger than 70 years. Amidst these data and the high prevalence of statin intake in wealthy countries, the role of lipid-lowering therapies in OSCC patients deserves further evaluation in comprehensive randomized controlled clinical trials.

## Figures and Tables

**Table 1:** Clinicopathological characteristics of OSCC patients according to statin use in the matched-pair cohort (*n* = 359, matching variables are highlighted in bold). UICC 7th edition.

		Statin Use						
		No		Yes		Total		χ2
		N	%	N	%	N	%	*p*
Sex	Female	75	28.5%	23	24.0%	98	27.3%	0.391
	Male	188	71.5%	73	76.0%	261	72.7%	
**Age at diagnosis**	**50–59**	**76**	**28.9%**	**25**	**26.0%**	**101**	**28.1%**	**0.840**
	**60–69**	**106**	**40.3%**	**39**	**40.6%**	**145**	**40.4%**	
	**70+**	**81**	**30.8%**	**32**	**33.3%**	**113**	**31.5%**	
**CCI**	**0**	**72**	**27.4%**	**24**	**25.0%**	**96**	**26.7%**	**0.852**
	**1**	**62**	**23.6%**	**21**	**21.9%**	**83**	**23.1%**	
	**2**	**59**	**22.4%**	**21**	**21.9%**	**80**	**22.3%**	
	**3+**	**70**	**26.6%**	**30**	**31.3%**	**100**	**27.9%**	
Positive anamnesis smoking	No	72	27.4%	28	29.2%	100	27.9%	0.738
	Yes	191	72.6%	68	70.8%	259	72.1%	
Positive anamnesis alcohol	No	82	31.2%	41	42.7%	123	34.3%	0.042
	Yes	181	68.8%	55	57.3%	236	65.7%	
Acetylsalicylic acid use	No	190	72.2%	43	44.8%	233	64.9%	<0.001
	Yes	73	27.8%	53	55.2%	126	35.1%	
Metformine use	No	242	92.0%	83	86.5%	325	90.5%	0.111
	Yes	21	8.0%	13	13.5%	34	9.5%	
Anatomical site	Upper alveolus and gingiva & Hard palate	31	11.8%	10	10.4%	41	11.4%	0.860
	Tongue	84	31.9%	29	30.2%	113	31.5%	
	Buccal mucosa & Lower alveolus and gingiva & Floor of mouth	148	56.3%	57	59.4%	205	57.1%	
**UICC stage**	**I**	**83**	**31.6%**	**32**	**33.3%**	**115**	**32.0%**	**0.895**
	**II**	**49**	**18.6%**	**18**	**18.8%**	**67**	**18.7%**	
	**III**	**41**	**15.6%**	**17**	**17.7%**	**58**	**16.2%**	
	**IV**	**90**	**34.2%**	**29**	**30.2%**	**119**	**33.1%**	
Tumorsize	T1	105	39.9%	40	41.7%	145	40.4%	0.957
	T2	87	33.1%	29	30.2%	116	32.3%	
	T3	20	7.6%	7	7.3%	27	7.5%	
	T4	51	19.4%	20	20.8%	71	19.8%	
Cervical lymph node metastasis	N0	172	65.4%	63	65.6%	235	65.5%	0.231
	N1	34	12.9%	18	18.8%	52	14.5%	
	N2/3	57	21.7%	15	15.6%	72	20.1%	
Extranodal spread	No	60	22.8%	22	22.9%	82	22.8%	0.936
	Yes	31	11.8%	10	10.4%	41	11.4%	
	not applicable	172	65.4%	64	66.7%	236	65.7%	
**Grading**	**G1**	**25**	**9.5%**	**7**	**7.3%**	**32**	**8.9%**	**0.763**
	**G2**	**186**	**70.7%**	**68**	**70.8%**	**254**	**70.8%**	
	**G3/4**	**52**	**19.8%**	**21**	**21.9%**	**73**	**20.3%**	
**Lymphatic invasion**	**L0**	**217**	**82.5%**	**77**	**80.2%**	**294**	**81.9%**	**0.616**
	**L1**	**46**	**17.5%**	**19**	**19.8%**	**65**	**18.1%**	
**Vascular invasion**	**V0**	**252**	**95.8%**	**93**	**96.9%**	**345**	**96.1%**	**0.647**
	**V1**	**11**	**4.2%**	**3**	**3.1%**	**14**	**3.9%**	
Adjuvant therapy	No	153	58.2%	65	67.7%	218	60.7%	0.054
	Radiotherapy	80	30.4%	17	17.7%	97	27.0%	
	Radiochemotherapy	30	11.4%	14	14.6%	44	12.3%	
	Total	263	100.0%	96	100.0%	359	100.0%	

Abbreviations: CCI: Charlson comorbidity index; UICC: Union for International Cancer Control; HR: hazard ratio; CI: confidence interval; OAS: overall survival; RFS: recurrence-free survival.

**Table 2:** Results from uni- and multivariable Cox regression analyses for overall survival in OSCC patients depending on statin use in complete cohort (*n* = 602). UICC 7th edition.

		Univariable Cox Regression				Multivariable Cox Regression			
			95%-CI				95%-CI		
		*p*	HR	Lower	Upper	*p*	HR	Lower	Upper
Statin use	No		1.000				1.000		
	Yes	0.418	0.869	0.619	1.220	**0.023**	**0.664**	**0.467**	**0.945**
Sex	Female		1.000						
	Male	0.844	0.974	0.746	1.271				
**Age at diagnosis**	**50–59**	<0.001 *				**0.059**	1.000		
	**60–69**	**0.374**	**1.139**	**0.855**	**1.519**	**0.820**	**1.035**	**0.773**	**1.385**
	**70+**	**<0.001**	**1.902**	**1.418**	**2.552**	**0.027**	**1.417**	**1.041**	**1.929**
**CCI**	**0**	**<0.001**	1.000			**<.001**	1.000		
	**1**	**0.064**	**1.357**	**0.983**	**1.872**	**0.063**	**1.362**	**0.983**	**1.887**
	**2**	**0.002**	**1.757**	**1.236**	**2.499**	**0.001**	**1.814**	**1.262**	**2.606**
	**3+**	**<.001**	**2.590**	**1.892**	**3.544**	**<.001**	**2.413**	**1.734**	**3.357**
Positive anamnesis smoking	No		1.000						
	Yes	0.551	1.090	0.822	1.445				
Positive anamnesis alcohol	No		1.000						
	Yes	0.784	1.037	0.799	1.346				
Acetylsalicylic acid use	No		1.000						
	Yes	0.223	1.179	0.905	1.536				
Metformine use	No		1.000						
	Yes	0.966	0.989	0.596	1.642				
Anatomical site	Upper alveolus and gingiva & Hard palate	0.495	1.000						
	Tongue	0.428	0.833	0.530	1.309				
	Buccal mucosa & Lower alveolus and gingiva & Floor of mouth	0.950	0.987	0.659	1.480				
**UICC stage**	**I & II**		1.000				1.000		
	**III & IV**	**<0.001**	**2.288**	**1.782**	**2.939**	**<0.001**	**2.054**	**1.586**	**2.660**
**Grading**	**G1**		1.000				1.000		
	**G3/4**	**0.065**	**1.326**	**0.983**	**1.789**	**0.324**	**1.170**	**0.856**	**1.598**
**Lymphatic invasion**	**L0**		1.000				1.000		
	**L1**	**<0.001**	**2.277**	**1.689**	**3.071**	**<0.001**	**1.763**	**1.286**	**2.415**
**Vascular invasion**	**V0**		1.000				1.000		
	**V1**	**0.001**	**2.339**	**1.410**	**3.881**	**0.231**	**1.387**	**0.812**	**2.368**
Adjuvant therapy	No	**<0.001**	1.000						
	Radiotherapy	**<0.001**	1.754	1.353	2.273				
	Radiochemotherapy	0.058	1.430	0.989	2.068				

Abbreviations: CCI: Charlson comorbidity index; UICC: Union for International Cancer Control; HR: hazard ratio; CI: confidence interval; OAS: overall survival; RFS: recurrence-free survival. Multivariable analysis adjusted for age, CCI, UICC stage, grading, lymphatic and vascular invasion, which proved to have *p* < 0.100 in univariable analysis. Tumor size, cervical lymph node metastasis and extranodal spread were dismissed in favor of UICC stage. Adjuvant therapy was not regarded as a confounder in this context (variables selected for multivariable analysis and for propensity score matching are highlighted in bold). * *p*-value in line of reference group denotes significance of the whole variable’s effect.

**Table 3:** Synopsis of results from uni- and multivariable Cox regression analyses for outcome in OSCC patients depending on statin use in complete and matched-pair cohort. UICC 7th edition.

		Univariable Cox Regression				Multivariable Cox Regression			
		*p*	HR	Lower 95%-CI	Upper 95%-CI	*p*	HR	Lower 95%-CI	Upper 95%-CI
Complete cohort (*n* = 602)	OAS	0.418	0.869	0.619	1.220	**0.023**	**0.664**	**0.467**	**0.945**
	Recurrence rate	0.108	0.634	0.364	1.106	0.067	0.588	0.334	1.037
	RFS	0.282	0.839	0.610	1.155	**0.014**	**0.662**	**0.476**	**0.920**
Complete cohort age 50–70 (*n* = 459)	OAS	**0.032**	**0.595**	**0.370**	**0.956**	**0.001**	**0.440**	**0.270**	**0.719**
	Recurrence rate	0.124	0.583	0.293	1.159	0.069	0.521	0.258	1.052
	RFS	**0.052**	**0.656**	**0.429**	**1.003**	**0.002**	**0.500**	**0.323**	**0.773**
Matched-pair cohort (*n* = 359)	OAS	0.100	0.740	0.517	1.059	**0.048**	**0.691**	**0.479**	**0.997**
	Recurrence rate	0.132	0.637	0.354	1.145	0.103	0.612	0.339	1.104
	RFS	0.075	0.736	0.525	1.032	**0.036**	**0.694**	**0.493**	**0.976**
Matched-pair cohort age 50–70 (*n* = 246)	OAS	**0.015**	**0.537**	**0.325**	**0.887**	**0.003**	**0.461**	**0.275**	**0.774**
	Recurrence rate	0.194	0.615	0.295	1.280	0.098	0.533	0.253	1.124
	RFS	**0.034**	**0.613**	**0.390**	**0.964**	**0.007**	**0.530**	**0.333**	**0.841**

Abbreviations: CCI: Charlson comorbidity index; UICC: Union for International Cancer Control; HR: hazard ratio; CI: confidence interval; OAS: overall survival; RFS: recurrence-free survival. For each subgroup an analysis for patients aged under 70 years is added. Multivariable analyses adjusted for age, CCI, UICC stage, grading, lymphatic and vascular invasion. Matching variables are highlighted in bold.

## Data Availability

Data available on request due to restrictions, e.g., privacy or ethical.
